# HBO1 catalyzes lysine benzoylation in mammalian cells

**DOI:** 10.1016/j.isci.2022.105443

**Published:** 2022-10-26

**Authors:** Doudou Tan, Wei Wei, Zhen Han, Xuelian Ren, Cong Yan, Shankang Qi, Xiaohan Song, Y. George Zheng, Jiemin Wong, He Huang

**Affiliations:** 1 Shanghai Institute of Materia Medica, Chinese Academy of Sciences, Shanghai, 201203, China; 2 Shanghai Key Laboratory of Regulatory Biology, Institute of Biomedical Sciences and School of Life Sciences, East China Normal University, Shanghai 200241, China; 3 Department of Pharmaceutical and Biomedical Sciences, University of Georgia, Athens, GA 30602, USA; 4 University of Chinese Academy of Sciences, Beijing 100049, China

**Keywords:** Biological sciences, Molecular biology, Cell biology

## Abstract

Lysine benzoylation (Kbz) is a newly discovered protein post-translational modification (PTM). This PTM can be stimulated by benzoate and contributes to gene expression. However, its regulatory enzymes and substrate proteins remain largely unknown, hindering further functional studies. Here we identified and validated the lysine acetyltransferase (KAT) HBO1 as a “writer” of Kbz in mammalian cells. In addition, we report the benzoylome in mammalian cells, identifying 1747 Kbz sites; among them at least 77 are the HBO1-targeted Kbz substrates. Bioinformatics analysis showed that HBO1-targeted Kbz sites were involved in multiple processes, including chromatin remodeling, transcription regulation, immune regulation, and tumor growth. Our results thus identify the regulatory elements of the Kbz pathway and reveal the non-canonical enzymatic activity and functions of HBO1 in cellular physiology.

## Highlights

• HBO1 is the “writer” of Kbz in mammalian cells
• HBO1 mainly exerts Kbz transferase activity at G485/E508
• 1747 Kbz sites were identified in mammalian cells
• HBO1-targeted Kbz sites are involved in diverse cellular processes

## Graphical abstract

**Figure:**
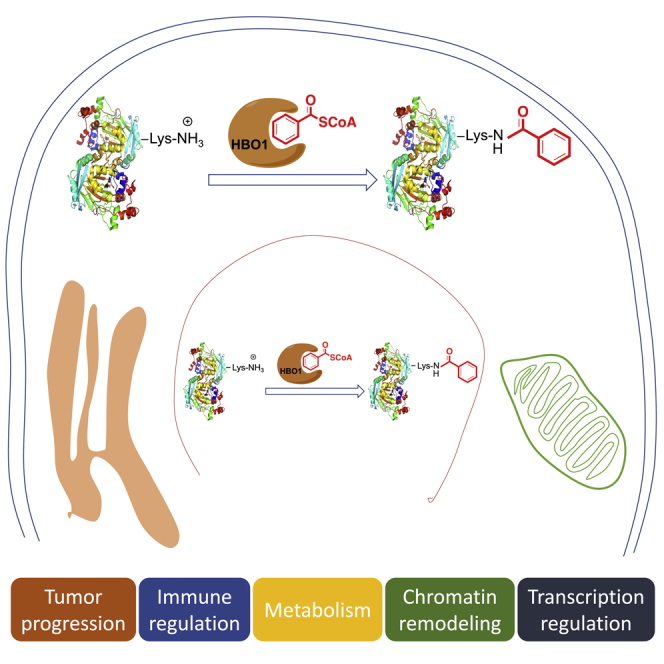

## Introduction

Post-translational modifications (PTMs) play important roles in diverse cellular processes, such as transcription, cell cycle, metabolism, and signal transduction (Dancy and Cole, 2015; Lundby et al., 2012; Sabari et al., 2017; Wang and Lin, 2021; Wang and Cole, 2020). Diverse lines of evidence suggest that aberrant PTMs contribute to many diseases (Husmann and Gozani, 2019; Lundby et al., 2019), and their regulatory enzymes, e.g., those for phosphorylation and lysine acetylation (Kac), represent an important class of protein targets for therapeutic drugs (Ferguson and Gray, 2018; Li and Ge, 2020). As has been demonstrated in many well-studied PTM pathways, knowledge of regulatory enzymes and substrate proteins is fundamental to the biochemical characterization of newly discovered PTMs, and offers a stepping stone to revealing their roles in physiology and pathology.

Recently, we discovered Kbz as a new type of physiologically relevant PTMs in mammalian cells and identified 22 histone Kbz marks (Huang et al., 2018c). We demonstrated that sodium benzoate (SB), a widely used food additive and a drug approved by the US Food and Drug Administration (FDA) for the treatment of hyperammonemia, could be converted to benzoyl-CoA in mammalian cells and served as the precursor of Kbz (Huang et al., 2018c). ChIP-seq and RNA-seq experiments showed that histone Kbz epigenetic marks are specifically located in promoter regions and are associated with gene expression (Huang et al., 2018c). However, the transferase enzymes that can catalyze Kbz in mammalian cells remain unknown. Moreover, non-histone Kbz substrates are likely to present in mammalian cells but their identities are not known. These knowledge gaps hinder the further functional characterization of this PTM pathway.

In this work, we revealed that HBO1, a KAT whose homolog does not exist in *S. cerevisiae*, acts as a “writer” of Kbz in mammalian cells. In addition, we report the global profiling of benzoylome in mammalian cells, identifying 1747 Kbz sites. Importantly, 77 of these Kbz sites are regulated by HBO1. This study thus discovered both the “writers” and protein substrates of Kbz in mammalian cells, significantly expanding our understanding of Kbz-regulated cellular cascades.

## Results and discussion

### HBO1 and HAT1 catalyze Kbz *in vitro*

**Figure 1:**
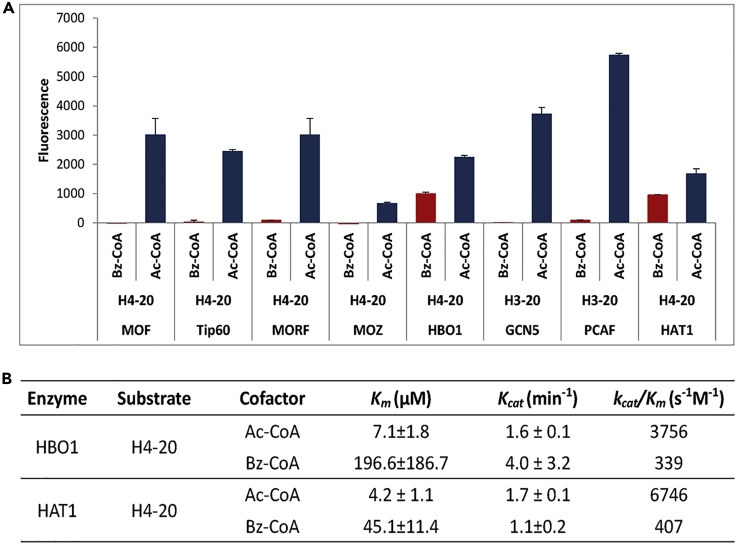
Screening of the Kbz transferases *in vitro* (A) Kac and Kbz catalytic activities of diverse KATs determined by fluorescence analysis. In the assay, the reaction mixture of benzoyl-CoA or acetyl-CoA was incubated with the synthetic H3-20 or H4-20 peptide substrate in the presence or absence of KAT enzymes at 30°C for 1 h. The fluorescence intensity of CoAS-CPM complex produced by the enzymatic reaction was measured by a microplate reader with the excitation and emission wavelength fixed at 392 and 482 nm. (B) Kinetics data of HBO1 and HAT1 toward catalyzing Kbz and Kac. In the assay, H4-20 peptide and HAT1 or HBO1 were co-incubated with acetyl-CoA or benzoyl-CoA at varying concentrations for 30 min at 30°C. The fluorescent CoAS-CPM complex was measured in the same way as the single-point fluorogenic assay. The kinetic constants *K*_*m*_ and *k*_*cat*_ were determined with the Michaelis-Menten model. Data are represented as means ±SEM (n = 3).

KATs are a group of enzymes that can catalyze Kac reactions on both histone and non-histone proteins (Huang et al., 2018a; Menzies et al., 2016). Emerging lines of evidence demonstrated that some KATs can catalyze multiple types of Kac-independent lysine acylations, such as crotonylation, β-hydroxybutyrylation, lactylation, and isobutyrylation (Xiao et al., 2021; Xie et al., 2016; Zhang et al., 2019; Zhu et al., 2021). Therefore, we hypothesized that some KATs may have benzoyltransferase activity as well. To test this hypothesis, we took advantage of an *in vitro* fluorometric KATs-catalyzed acylation assay wherein benzoyl-CoA and synthetic human histone H3 or H4 peptides are used as co-factor and substrate, respectively (Gao et al., 2013b). Using this assay, we screened eight KATs, including MOF, Tip60, MOZ, MORF, HBO1, GCN5, PCAF, and HAT1. As expected, all the KATs showed good acetyltransferase activities. On the other hand, only HBO1 and HAT1 showed significant catalytic activities for Kbz (Figure 1A).

We next sought to quantitatively compare the acyltransferase activities of HAT1 and HBO1 in the Kac and Kbz reactions. The kinetic analysis results showed that the *K*_*cat*_ values of HAT1 and HBO1 in the Kbz reaction were close to those for Kac. However, the *K*_*cat*_/*K*_*m*_ values of HBO1 and HAT1 were 339s^−1^M^−1^ and 407s^−1^M^−1^ for Kbz, respectively, which were 9 and 6% of their *K*_*cat*_/*K*_*m*_ values for Kac (Figure 1B). These biochemical results indicated that HBO1 and HAT1 could catalyze Kbz *in vitro*, although their catalytic activities for Kbz were not as good as for Kac.

### HBO1 catalyzes Kbz in mammalian cells

**Figure 2:**
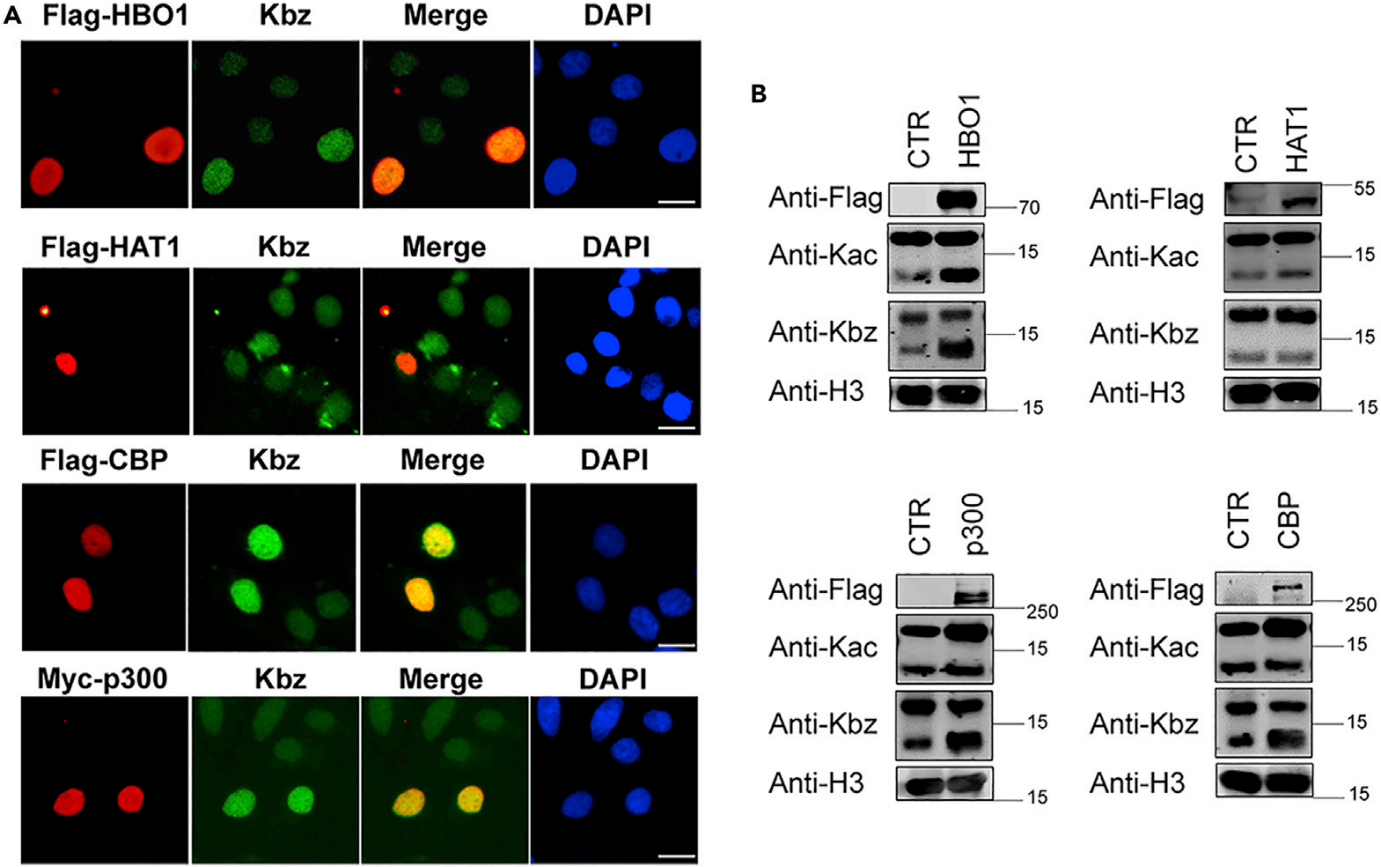
Evaluation of the candidate Kbz transferases in mammalian cells (A) Immunofluorescence staining determines the Kbz levels in response to the overexpression of HBO1, HAT1, p300, and CBP in HeLa cells. HeLa cells were washed with PBS, fixed with 4% paraformaldehyde, incubated on ice with Triton X-100, blocked by 5% BSA, and co-incubated with primary antibody for 2 h. The Texas Green conjugated secondary antibody against mouse or rabbit was incubated. Images were acquired with an Olympus microscope system. Scale bar = 10 μm. (B) Western blot analysis determines the Kbz levels in response to the overexpression of HBO1, HAT1, p300, and CBP in HeLa cells. The recombinant plasmids were transiently transfected into HeLa cells. Histones purified from cells were used to detect the indicated marks by western blot.

To evaluate the cellular benzoyltransferase activity of HBO1 and HAT1, we performed immunofluorescence (IF) staining of Kbz in response to the overexpression of diverse KATs in HeLa cells. Given the broad acyltransferase activity of p300 and CBP (Dancy and Cole, 2015; Huang et al., 2018b), we also include them in the analysis. As expected, ectopic expression of these KATs led to a substantial increase in Kac. Notably, the Kbz levels increased significantly in the cells overexpressing HBO1, p300, and CBP (Figure 2A). However, no obvious change in the Kbz levels could be detected in response to the overexpression of HAT1, GCN5, MOF, PCAF, and Tip60 (Figures 2A and S1).

To confirm the Kbz transferase activity of HBO1, CBP, and p300 in cells, we overexpressed them in 293T cells and determined the Kbz levels by western blot (WB). Consistent with the IF results, WB analysis showed that overexpression of HBO1, p300, and CBP, but not HAT1, led to increased histone Kbz levels (Figure 2B). It is not surprising that p300 and CBP catalyzed Kbz in cells because their catalytic pockets are large enough to accommodate diverse acyl-CoAs (Liu et al., 2008). Taken together, our results demonstrate that HBO1 can catalyze Kbz both *in vitro* and in mammalian cells.

### Validating the Kbz transferase activity of HBO1

**Figure 3:**
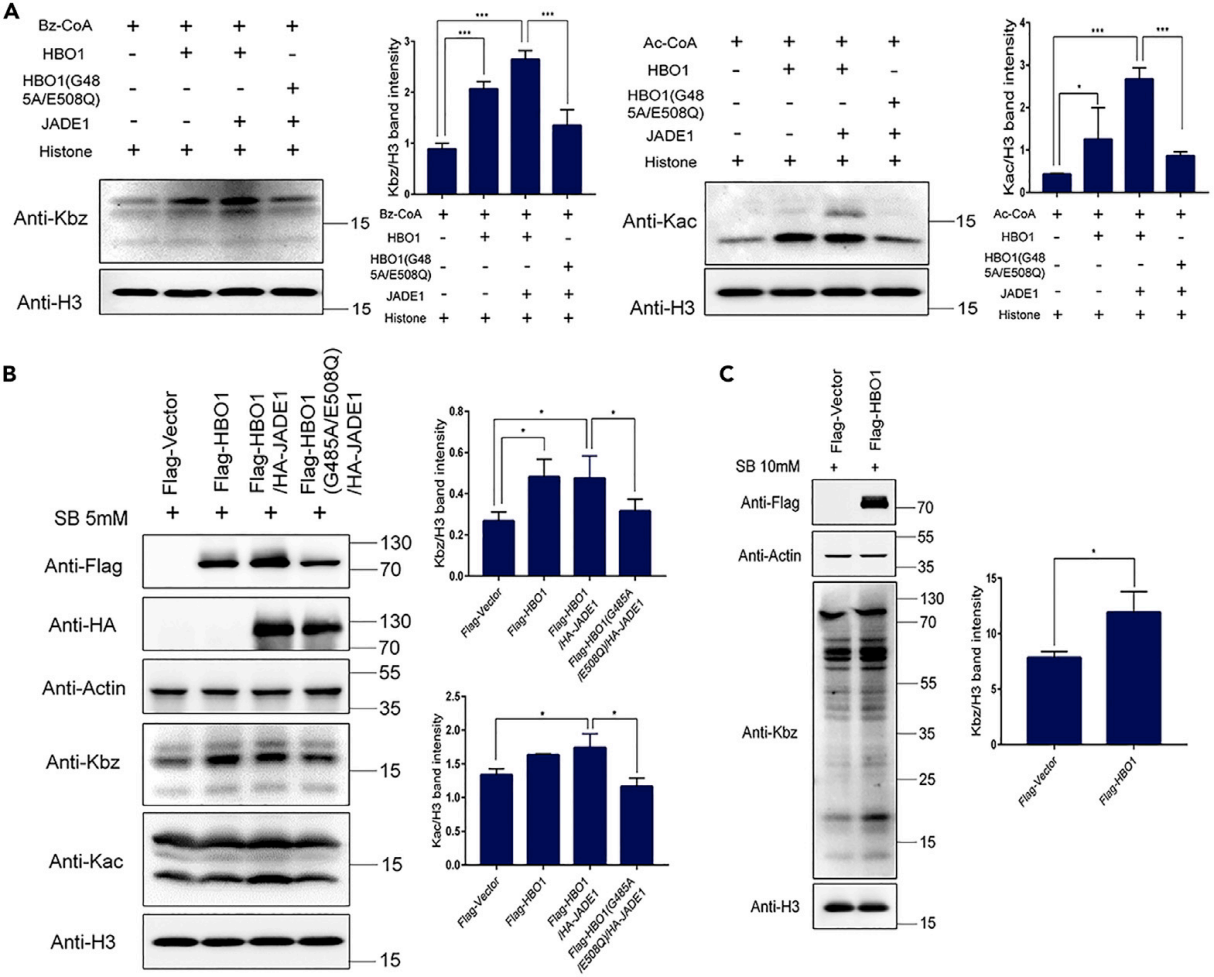
HBO1 catalyzes Kbz both *in vitro* and in mammalian cells (A) HBO1 catalyzes Kac and Kbz reactions *in vitro*. Kbz or Kac activities of HBO1 WT/MUT were detected using histones as substrate. Reaction products were detected by western blot with indicated antibodies. (B) HBO1 catalyzes histone Kbz in mammalian cells. WT and MUT HBO1 were overexpressed alone or in combination with JADE-1 in 293T cells (incubating with 5mM of SB at 24 h after transfection). Whole-cell lysates were collected at 48 h after transfection. Levels of Kbz and Kac were detected by western blot and H3 served as a loading control. (C) HBO1 catalyzes global Kbz in 293T cells. WT HBO1 was overexpressed in 293T cells and the changes of global Kbz levels were detected by western blot.Data are represented as mean ± SEM, ∗pvalue less than 0.05; ∗∗pvalue less than 0.01; ∗∗∗pvalue less than 0.001.

To validate the benzoyltransferase activity of HBO1, we first carried out the Kbz reactions *in vitro* using core histones extracted from 293T cells and benzoyl-CoA as substrate and co-factor, respectively. Acetyl-CoA was used as a positive control for the assay. WB results showed that wild-type (WT) HBO1 could increase both the Kbz and Kac levels in the core histones (Figure 3A). In addition, its transferase activity was enhanced with the presence of the scaffold protein JADE-1. In contrast, the enzyme-dead mutation (MUT) of HBO1 (G485A/E508Q) abolished its Kbz and Kac transferase activities (Figure 3A). Consistent with the *in vitro* assay results, when WT HBO1 was overexpressed alone or in combination with JADE-1 in 293T cells, the Kbz levels were increased (Figure 3B). However, no obvious changes in the Kbz level could be detected when the MUT HBO1 (G485A/E508Q) was overexpressed (Figure 3B).

Next, to investigate whether HBO1 regulates non-histone Kbz in mammalian cells, we overexpressed WT HBO1 in 293T cells and detected the changes in global Kbz levels. The results showed that overexpression of WT HBO1 elevated the Kbz levels of both non-histone and histone proteins (Figure 3C). All the evidence indicates that Kbz transferase activity is indeed indigenous to HBO1, and it mediates Kbz globally.

**Figure 4:**
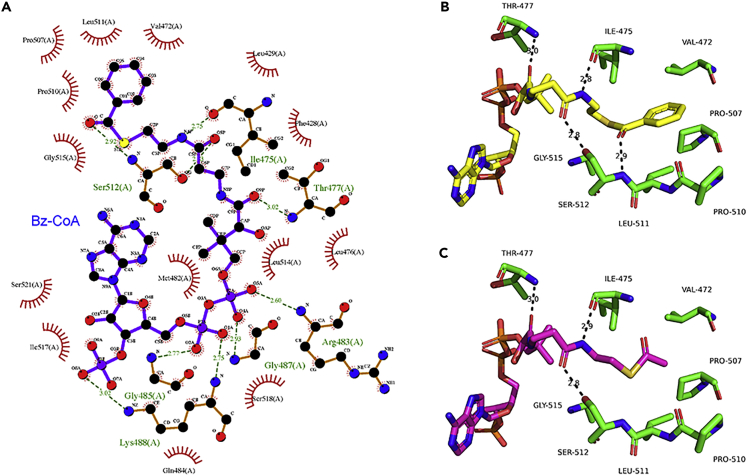
Molecular docking predicts the binding mode of benzoyl-CoA to HBO1 (A) Ligplot analysis (Laskowski and Swindells, 2011) shows the interactions between HBO1 and benzoyl-CoA. HBO1 protein was extracted from the crystal structure of human HBO1 in complex with acetyl-CoA, and the benzoyl-CoA was generated based on the structure of acetyl-CoA using the PyMol package. (B) Predicted binding mode of benzoyl-CoA to HBO1. Molecular docking was performed using the AutoDock program. The results were evaluated by clustering analysis and visual inspection. (C) Predicted binding mode of acetyl-CoA to HBO1.

In addition, molecular docking predicted that the benzoyl-CoA could interact with Thr477, Ile475, Leu511, and Ser512 residues of HBO1 through hydrogen bond interactions, which is similar to acetyl-CoA (Figure 4A). Moreover, a large hydrophobic pocket consisting of Val472, Pro507, Pro510, Leu511, and Gly515 may further stabilize the benzoyl moiety by hydrophobic interactions (Figures 4B and 4C).

### Quantitative benzoylome expands the roles of HBO1 and Kbz

**Figure 5:**
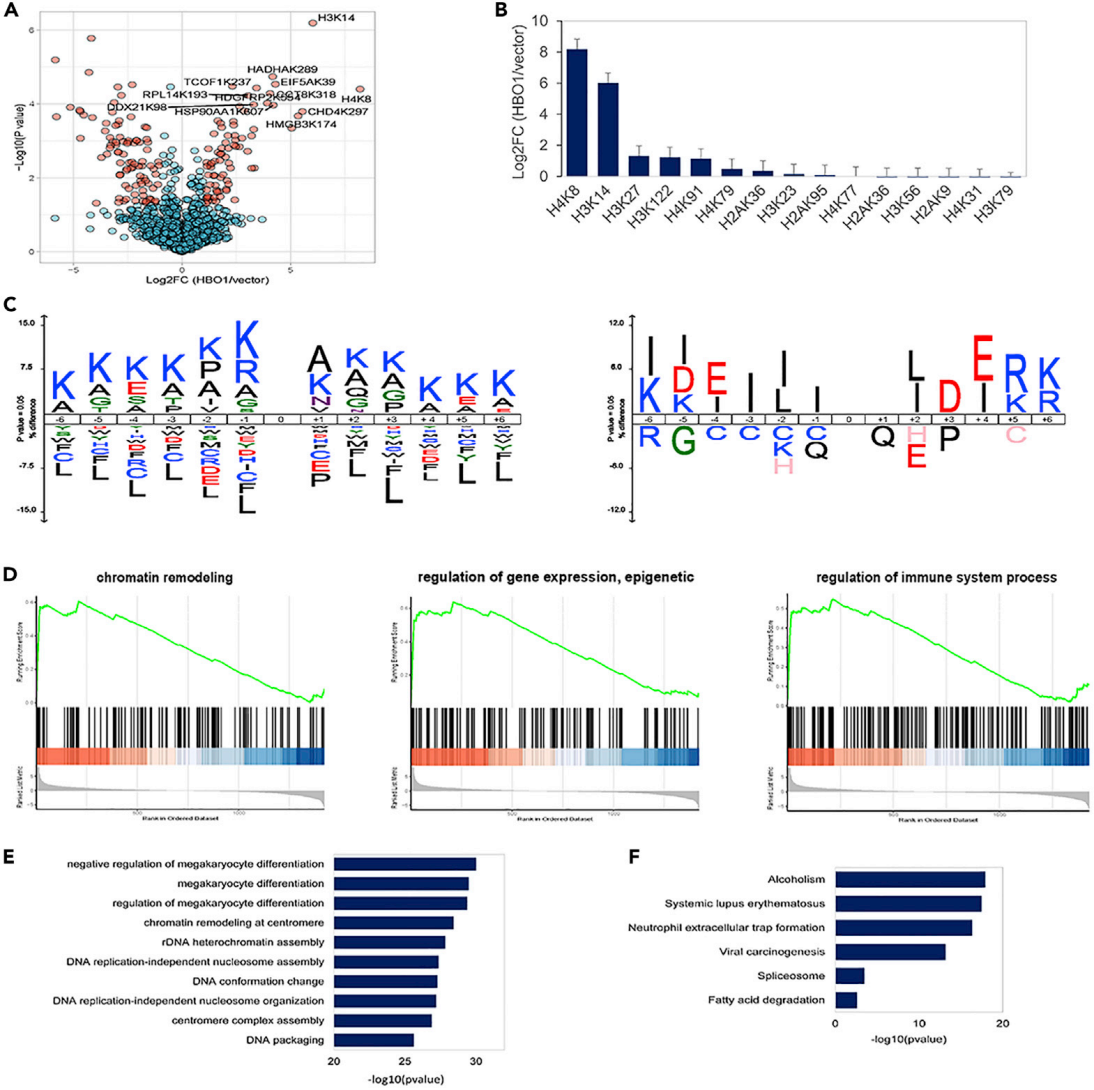
HBO1-mediated Kbz is associated with diverse cellular processes (A) Volcano map shows the dynamics of Kbz sites in response to HBO1 overexpression. Quantitative proteomic analysis was performed with three biological replicates in response to HBO1 overexpression in 293T cells. When log2 (overexpression/control) > 1 and p < 0.05, the quantified Kbz sites were considered significantly upregulated. (B) Dynamics of histone Kbz sites in response to HBO1 overexpression. Histograms show significantly upregulated core histone sites. Data are represented as mean ±SEM. (C) Motifs of the residues around the Kbz sites identified in mammalian cells (left) and reported in *S. cerevisiae* (right), respectively. (D–F) (D) GO-MF term GSEA, (E) GO-BP term enrichment, and (F) KEGG pathway analysis of the proteins bearing HBO1-targeted Kbz sites.

Given that HBO1 mediates Kbz globally in mammalian cells, we next asked which Kbz protein substrate and cellular processes are targeted by HBO1. To this end, we performed a quantitative proteomics analysis with three biological replicates to identify the global Kbz sites and quantify their dynamics in response to HBO1 overexpression in 293T cells. In total, 1747 Kbz sites were identified and 1344 of them were quantified (Table S1). The changes of each Kbz site were normalized by the dynamics of corresponding protein levels. Among the quantified Kbz sites, 77 of them were significantly upregulated (log2(overexpression/control) > 1 and p< 0.05) and served as the HBO1-targeted Kbz substrates (Figure 5A). Of interest, H4K8bz and H3K14bz increased by 292.8 and 63.9-folds, respectively (Figure 5B), suggesting that HBO1 may exert epigenetic functions through the regulation of H4K8bz and H3K14bz. These results are reasonable because H4K8 and H3K14 are known major target substrate sites of HBO1 (Tao et al., 2017).

Recently, it was reported that the GCN5-containing complex can serve as a histone benzoyltransferase in *S. cerevisiae* (Wang et al., 2022). However, our results showed that its homolog GCN5 could not catalyze the Kbz reaction in mammalian cells. Moreover, the HBO1 homolog does not exist in yeast. Therefore, we hypothesized that the flanking sequence motifs of Kbz in mammalian cells and yeast are quite different. In support of this notion, the positively charged amino acids Lys and Arg were over-represented and the hydrophobic amino acid Leu was largely depleted in mammalian cells, whereas the hydrophobic and negatively charged amino acids Leu, Ile, Asp, and Glu were enriched in *S. cerevisiae* (Figure 5C).

Gene set enrichment analysis (GSEA) of the Gene Ontology Molecular Function (GO-MF) term indicated that many non-histone Kbz proteins were involved in transcriptional regulation, such as Chromatin remodeling (adjusted p = 2.55E-02) and Regulation of gene expression (adjusted p = 1.80E-03) (Figure 5D). GO Biological Processes (BP) term enrichment analysis of the proteins bearing HBO1-targeted Kbz sites suggested that HBO1 may play important roles in Megakaryocyte differentiation (adjusted p = 2.59E-27), Chromatin remodeling at centromere (adjusted p = 1.76E-26), and Nucleosome assembly (adjusted p = 1.35E-25) through the Kbz pathway (Figure 5E). In addition, the Kyoto Encyclopedia of Genes and Genomes (KEGG) pathway analysis suggested that HBO1-targeted Kbz proteins were associated with diverse physiological functions, such as Alcoholism (adjusted p = 1.29E-16), Spliceosome (adjusted p = 6.75E-03), and Fatty acid degradation (adjusted p = 4.37E-02) (Figure 5F). These results suggest that HBO1 may regulate diverse cellular physiological and pathological processes by mediating the global benzoylome.

Kbz may also affect the cell fate by regulating the functions of key proteins. For example, chromodomain helicase DNA-binding 4 (CHD4), which contains 5 Kbz sites, was confirmed to play a crucial role in chromatin remodeling and regulation of gene expression (Weiss et al., 2020). In addition, Kbz may be associated with diseases such as cancer. B cell lymphoma-2-associated transcription factor 1 (BCLAF1), a death-promoting transcriptional repressor, participates in various biological processes, including autophagy and DNA damage response, and is highly associated with the proliferation and drug-resistance of cancer (Mou et al., 2020; Yu et al., 2022). Notably, it contains eight Kbz sites, therefore linking Kbz to tumor development.

Given that some benzoylated lysine residues could also be acetylated, next we systematically compared the benzoylome with known Kac sites based on the UniProt database. The results showed that only 268 Kbz sites could also be acetylated (Table S1), and the proteins with the overlapped sites were mainly involved in chromatin remodeling, gene expression regulation, and metabolism. Of interest, the proteins that only contained Kbz sites were associated with multiple human diseases, such as tumor progression, suggesting that Kbz has unique biological functions from Kac.

Emerging evidence indicates that different lysine acylations demonstrate unique cellular functions, even though they have similar structures. Compared with the Kac, Kbz has a larger and more hydrophobic moiety, and therefore, may differentially exert cellular functions based on its structural specificity (Ren et al., 2021). Although both Kac and Kbz are regulated by HBO1, acetyl-CoA and benzoyl-CoA may compete for the active site of HBO1 in the same cellular microenvironment, thus affecting the substrate selectivity of HBO1 and leading to different functional outcomes.

### Significance

In this study, we identified HBO1 as a Kbz transferase both *in vitro* and in mammalian cells. Moreover, we report a global benzoylome in mammalian cells, identifying 1747 Kbz sites and revealing at least 77 HBO1-targeted Kbz sites. Functional analysis showed that the HBO1-targeted Kbz sites were associated with diverse cellular physiological and pathological processes, such as chromatin remodeling, transcription regulation, metabolism, immune regulation, tumor progression, and so on. Excessive intake of SB can raise Kbz levels and increase the risk of some diseases, such as motor coordination impairment and ADHD symptoms. Given that HBO1 serves as a Kbz transferase in mammalian cells, our findings thus provide a potential strategy for treating these diseases through the regulation of the Kbz by interfering with HBO1.

### Limitations of the study

In this study, we identified lysine acetyltransferase HBO1 as the “writer” of Kbz in mammalian cells. In addition, we report 1747 Kbz sites in mammalian cells, and at least 77 are HBO1-targeted Kbz substrates. Although bioinformatics analysis revealed that HBO1-targeted Kbz sites are involved in a variety of cellular physiological and pathological processes, these functional sites still require extensive validation at the cellular and animal levels. Further, the roles of some Kbz sites in important proteins remain to be investigated, which may provide some insights for the treatment of certain diseases.

## Additional resources

This article does not report additional websites or resources.

## STAR★Methods

### Key resources table

**Table:** 

REAGENT or RESOURCE	SOURCE	IDENTIFIER
**Antibodies**		

Monoclonal ANTI-FLAG M2 antibody	Sigma-Aldrich	Cat# F3165; RRID:AB_259529
ANTI-FLAG M2 Affinity Gel	Sigma-Aldrich	Cat# A2220; RRID:AB_10063035
Myc-Tag Mouse mAb	Cell Signaling Technology	Cat# 2276; RRID:AB_331783
Anti-Histone H3 antibody	Huabio	Cat# M1306-4
Beta Actin Monoclonal antibody	Proteintech	Cat# 66009-1-Ig; RRID:AB_2687938
Anti-Benzoyllysine Rabbit mAb	PTM Biolabs	Cat# PTM-761
Anti- Benzoyllysine antibody conjugated agarose beads	PTM Biolabs	Cat# PTM-763
Anti-Acetyllysine Mouse mAb	PTM Biolabs	Cat# PTM-101

**Chemicals, peptides, and recombinant proteins**		

H3-20 peptide: Ac-ARTKQTARKSTGGKAPRKQL	This paper	N/A
H4-20 peptide: Ac-SGRGKGGKGLGKGGAKRHRK	This paper	N/A
Benzoyl coenzyme A lithium salt	Sigma-Aldrich	Cat# B1638
Acetyl coenzyme A lithium salt	Sigma-Aldrich	Cat# A2181
Critical commercial assays		
lipofectamine 2000	Invitrogen	Cat# 11668019

**Deposited data**		

Proteomics dataset of the Kbz sites in the quantitative benzoylome	PRIDE	PXD034187

**Experimental models: Cell lines**		

293T	NCACC	Cat# GNHu17
HepG2	NCACC	Cat# TCHu72
Hela	NCACC	Cat# TCHu187

**Recombinant DNA**		

Plasmid: pCMV-Flag-HAT1	This paper	N/A
Plasmid: pCMV-Flag-CBP	This paper	N/A
Plasmid: pCMV-Myc-p300	This paper	N/A
Plasmid: pCMV-Flag-GCN5	This paper	N/A
Plasmid: pCMV-Flag-MOF	This paper	N/A
Plasmid: pCMV-Flag-PACF	This paper	N/A
Plasmid: pCMV-Flag-Tip60	This paper	N/A
Plasmid: pCMV-Flag-HBO1	This paper	N/A
Plasmid: pCMV-Flag-HBO1 G485A/E508Q	This paper	N/A
Plasmid: pCMV-Flag-JADE1	This paper	N/A

**Software and algorithms**		

MaxQuant software (v1.6.15.0)	MaxQuant	https://www.maxquant.org/
PyMol package	Pymol	http://www.pymol.org/
R clusterProfiler package	R-project	https://www.r-project.org/
iceLogo (v1.2)	IceLogo	https://iomics.ugent.be/icelogoserver/
ImageJ	National Institutes of Health	https://imagej.nih.gov/ij/

### Resource availability

#### Lead contact

Further information and requests for resources and reagents should be direct to and will be fulfilled by the Lead Contact, He Huang (hhuang@simm.ac.cn).

#### Materials availability

The plasmids used in this study are listed in the key resources table and are available from the lead contact on request.

### Experimental model and subject details

#### Cell lines

HepG2, Hela, and 293T cell lines were purchased from the National Collection of Authenticated Cell Cultures (NCACC) and used without further authentication. Cells were cultured in high-glucose DMEM supplemented with 10% FBS and incubated at 37°C under 5% CO_2_. No mycoplasma contamination was detected using the reported method (Dreolini et al., 2020).

### Method details

#### Reagents

Unless otherwise noted, all chemical reagents were purchased from Sangon Biotech (Shanghai) Co., Ltd. Antibodies were the following: anti-Kac (1:1000, PTM Biolabs, PTM-101), anti-H3 (1:10,000, Huabio, M1306-4), anti-Actin (1:10,000, Proteintech, 66009-1-Ig), anti-Flag (1:10,000; Sigma-Aldrich, F3165), anti-Myc (1:10,000; Cell Signaling Technology, 2276), anti-Kbz antibody (1:1000, PTM Biolabs, PTM-761), pan anti-Kbz beads (PTM Biolabs, PTM-763), and anti-Flag M2 Affinity Gel (Sigma-Aldrich, A2220).

#### *In vitro* screening of the acyltransferase activities of KATs

The benzoylation activity of the KATs was first screened with a fluorogenic assay (Gao et al., 2013a). Synthetic H3-20 or H4-20 peptides containing 20 amino acid residues from the N-terminal of histone H3 and H4 were used as the acyl acceptors. The sequences of H3-20 and H4-20 are Ac-ARTKQTARKSTGGKAPRKQL and Ac-SGRGKGGKGLGKGGAKRHRK, respectively. In the assay, 30 mL of reaction mixture containing 18 mM benzoyl-CoA (Sigma-Aldrich, B1638) or acetyl-CoA (Sigma-Aldrich, A2181), 100 mM peptide substrates, and 100 nM enzymes in the reaction buffer containing 50 mM HEPES, pH 8.0 and 0.1 mM EDTA was incubated at 30°C for 1 h. After the incubation, 30 μL of dimethyl sulfoxide (DMSO) solution containing 50 mM 7-diethylamino-3-(4′-maleimidylphenyl)-4-methylcoumarin (CPM) were mixed with the reaction mixture, followed by co-incubation in darkness at room temperature for 20 min. Addition of DMSO solution quenched the enzymatic reaction and the CPM reacted with the by-product Coenzyme A (CoA-SH) to produce the fluorescent CoA-CPM complex. The fluorescence intensity was then measured with a microplate reader (FlexStation 3) with the excitation and emission wavelength fixed at 392 and 482 nm. The negative control samples were treated in the same way except that the reaction buffer was added to substitute KAT enzymes. Duplicate experiments were performed and the results were summarized in Figure 1A.

The benzoylation activity of HBO1 and HAT1 was further characterized with a kinetic assay used in a previous study (Han et al., 2017). In the assay, 200 mM of H4-20 peptide and 100 nM of HAT1 or HBO1 were co-incubated with acetyl-CoA or benzoyl-CoA at varying concentrations for 30 min at 30°C in the reaction buffer containing 50 mM HEPES, pH 8.0 and 0.1 mM of EDTA. 50 mM CPM in DMSO solution was added to quench the enzymatic reaction and to produce the fluorescent CoAS-CPM complex. The fluorescence intensity was measured in the same way as the single-point fluorogenic assay. The kinetic constants *K*_*m*_ and *k*_*cat*_ were determined with the Michaelis-Menten model and were summarized in Figure 1B.

#### Transfections, immunofluorescent staining

DNA transient transfection was performed using lipofectamine 2000 (Invitrogen, 11,668,019) according to the manufacturer’s instructions. Immunofluorescence staining and western blot for various proteins were carried out essentially as described (Liu et al., 2013). For immunofluorescence staining, HeLa cells were washed with 1xPBS (137mM NaCl, 2.7mM KCl, 10mM Na_2_HPO_4_, and 2mM KH_2_PO_4_) before fixation in 4% paraformaldehyde at room temperature for 20 min, incubated with 1% Triton X-100 on ice for 15 min, blocked with 5% BSA in 37°C incubator for 60 min and incubated with the mouse or rabbit anti-Flag/Myc antibody for 2h. The coverslips were washed 3 times with PBST, followed by incubation with Texas Green conjugated secondary antibody against mouse or rabbit. Images were acquired with an Olympus microscope system.

#### Western blot analysis

Histones were purified from cells using a standard acid extraction protocol (Shechter et al., 2007). The protein extract (20 μg whole cell protein or 4 μg histone) was fractionated by SDS-PAGE electrophoresis and transferred to the PVDF membrane (GE) using a transfer device according to the manufacturer’s protocol (Biotanon, VE-186). After incubating with 3% BSA in TBST (10 mM Tris, pH 8.0, 150 mM NaCl, 0.5% Tween 20) for 1 h, incubate the membrane with the designated primary antibody (the concentration is shown in the “Reagents” section) overnight at 4°C. Then the membrane was washed 3 times with TBST (5 min each time), and horseradish peroxidase-conjugated anti-mouse or anti-rabbit antibody (1:20,000, Jackson, 115-035-146/111-035-144) was incubated for 1 h at room temperature. Next, the membrane was washed 3 times with TBST (5 min each time) and developed using a chemiluminescence detection system (Biotanon, 4600) according to the manufacturer’s protocol.

#### Immunoaffinity purification of HBO1 and complexes from HEK293T cells

Plasmids encoding wild-type or mutant Flag-HBO1 were transfected into 293T cells with or without the plasmid encoding Flag-JADE-1. Cells were collected at 48 h after transfection. Then, the cells were washed with precooled PBS and lysed with lysis buffer (50 mM Tris-HCl pH 7.5, 150 mM sodium chloride, Triton X-100 1%, 1 mM EDTA, 1 mM DTT, 8% glycerol plus protease inhibitor) on ice for 30 min. After centrifugation at 13,400gat 4°C for 10 min, the supernatant was collected and incubated with 10 μL of Flag-M2 beads at 4°C for 2 h. After incubation, the supernatant was discarded and the Flag-M2 beads are washed 3 times with washing buffer (20 mM Tris-HCl pH7.5, 150 mM NaCl, 0.1% Triton X-100, 1 mM EDTA, 1 mM DTT, 8% glycerol plus protease inhibitor). Next, the target protein was eluted by elution buffer (20 mM Tris-HCl pH7.5, 150 mM NaCl, 0.1% NP-40, 1 mM DTT, 10% glycerin plus protease inhibitors).

#### *In vitro* histone acylation assay

Histones were extracted from 293T cells using a standard acid extraction protocol (Shechter et al., 2007). The reaction mixtures (including 100 μM of benzoyl-CoA or acetyl-CoA, 2 μg of enzymes as indicated, 100 nM TSA, and 4 μg extracted histone) were incubated in reaction buffer (25 mM Tris-HCl pH 8.0, 150 mM NaCl, 10% glycerol, 1 mM DTT) at 37°C for 1 h. After the incubation, 5×SDS loading buffer was added to the mixture to quench the reaction, and the levels of Kac and Kbz were determined by western blot.

#### Immunoprecipitation

The peptide samples in NH_4_HCO_3_ solution were incubated with 30 μL of pan anti-Kbz beads at 4°C overnight. After incubation, the beads were washed three times with NETN buffer (50mM Tris pH 8.0, 100mM NaCl, 1mM EDTA, 0.5% NP40), twice with ETN buffer (50mM Tris pH 8.0, 100mM NaCl, 1mM EDTA), and once with water. The bound peptides were eluted from the beads with 0.1% trifluoroacetic acid and vacuum-dried.

#### HPLC-MS/MS analysis of Kbz

The sample analysis was carried out on an EASY-nLC 1200 UHPLC system (ThermoFisher Scientific) coupled with a Q Exactive HF-X mass spectrometer (ThermoFisher Scientific). Peptides were dissolved in 2.5 μL of solvent A (0.1% FA in water, v/v) and injected into a homemade packed capillary C18 column (20 cm length×75 μm ID, 1.9 μm particle size, Dr. Maisch GmbH, Germany). The quantitative proteome and immunoprecipitated Kbz samples were run in 180- and 120-min gradient, respectively, from 6 to 90% solvent B (A, 0.1% formic acid; B, 80% acetonitrile in 0.1% formic acid). Full mass scans were acquired with 350–1200 *m*/*z* at a mass resolution of 60,000. Ions with 2+, 3+, and 4+ charge were selected for MS/MS analysis. The 12 most intensive ions were fragmented with 28% normalized collision energy and tandem mass spectra were acquired with a mass resolution of 15,000. Dynamic exclusion was set to 30 s. The AGC numbers were 3 × 10^6^ and 2 × 10^5^ for MS1 and MS2, respectively. The isolation window was set to 1.3 *m*/*z*.

#### Protein sequence database searching

After LC-MS/MS acquisition, the raw files were qualitatively analyzed by MaxQuant software (version1.6.15.0) against the UniProt human database (20,376 entries). Parameters set for quantitative proteomics identification include Trypsin/P as the digestive enzyme; maximum missing cleavage of 2; minimum peptide length of 7; maximum FDR for peptides and proteins of 1%. Cysteine carbamidomethylation was established as a fixed modification. Methionine oxidation and acetylation of the N-terminus were established as variable modifications. Parameter setting of Kbz samples included lysine benzoylation as a variable modification. Other parameters were consistent with the proteome search. FDR thresholds for modification sites were specified at 1%. All the Kbz site ratios were normalized by the quantified protein expression levels.

#### Molecular docking

HBO1 protein was extracted from the crystal structure of human HBO1 in complex with acetyl-CoA (PDB: 5GK9), and the benzoyl-CoA was generated based on the structure of acetyl-CoA using the PyMol package (http://www.pymol.org/). The docking files were prepared with AutoDockTools-1.5.6. The receptor grid file was generated with a box size of 126 × 100 × 100 to cover the protein and calculated by the Autogrid program. The genetic algorithm was selected as “search parameters” (the number of GA runs was set as 100 and other parameters were kept unchanged) and the “docking parameters” was set as default. Molecular docking was performed using the AutoDock program (Morris et al., 2009). The results were evaluated by clustering analysis and visual inspection.

#### Bioinformatics analysis

The quantitative proteomics experiments were performed with three biological replicates. All the Kbz site ratios were normalized by corresponding quantified protein expression levels. The quantitative Kbz proteome was analyzed by a two-tailed Student’s *t*test for the two groups with HBO1 or vector plasmids transfection. GO, KEGG, and GSEA analyses were adopted with a hypergeometric test in the R clusterProfiler package (Yu et al., 2012). The consensus sequence logo analysis was performed using iceLogo (v1.2) (Colaert et al., 2009).

### Quantification and statistical analysis

Experimental values are presented as mean ±SEM. The quantitative benzoylome data were analyzed by a two-tailed Student’s *t*test. Differential expression was considered to be significant when p< 0.05, ∗p value less than 0.05; ∗∗p value less than 0.01; ∗∗∗p value less than 0.001.

## Data Availability

• The mass spectrometry proteomics data have been deposited to the ProteomeXchange Consortium via the PRIDE partner repository with the dataset identifier PXD034187. The list of Kbz sites in the quantitative benzoylome is provided in Table S1. • This article does not report original code. • Any additional information required to reanalyze the data reported in this article is available from the lead contacton request.

## Supplemental information

Document S1. Figures S1

Table S1. List of the Kbz sites in the quantitative benzoylome, related to Figure 5
