# Supplementary material for: HBO1 catalyzes lysine benzoylation in mammalian cells
Source: iScience. 2022 Oct 26;25(11):105443. doi: 10.1016/j.isci.2022.105443 (PMC9647509; doi:10.1016/j.isci.2022.105443)
Supplement: Document S1. Figures S1 [file mmc1.pdf]

iScience, Volume *25*

Supplemental information

HBO1 catalyzes lysine benzoylation

in mammalian cells

Doudou Tan, Wei Wei, Zhen Han, Xuelian Ren, Cong Yan, Shankang Qi, Xiaohan Song, Y. George Zheng, Jiemin Wong, and He Huang

HBO1 Catalyzes Lysine Benzoylation in Mammalian Cells

DoudouTan[1], Wei Wei[2], Zhen Han[3], Xuelian Ren[1], Cong Yan[1], Shankang Qi[1], Xiaohan Song[1], Y. George Zheng[3], Jiemin Wong[2], He Huang[1,4,5*]

[1]**Shanghai Institute of Materia Medica, Chinese Academy of Sciences, Shanghai, 201203, China**

[2]Shanghai Key Laboratory of Regulatory Biology, Institute of Biomedical Sciences and School of Life Sciences, East China Normal University, Shanghai 200241, China

[3]Department of Pharmaceutical and Biomedical Sciences, University of Georgia, Athens, GA 30602, USA

[4]University of Chinese Academy of Sciences, Beijing, 100049, China

[5]Lead contact

[*]Correspondence: hhuang@simm.ac.cn

Supplemental Figure

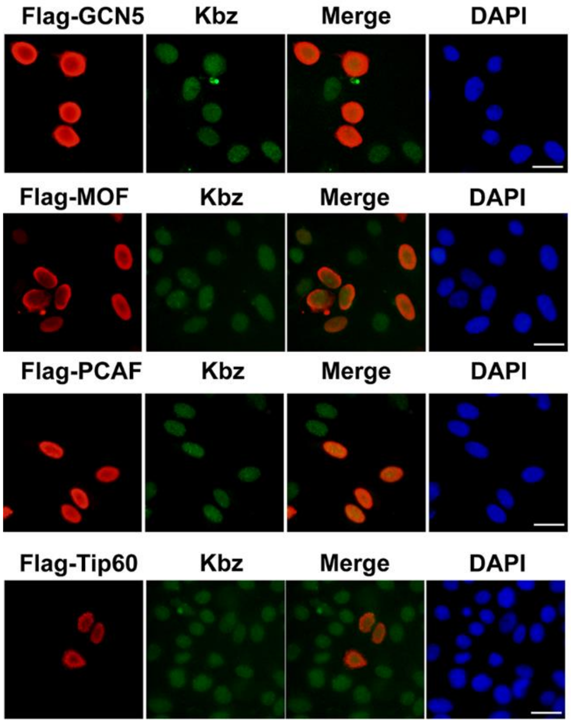

Figure S1. Related to Figure 2.

After GCN5, MOF, PCAF, and Tip60 were transfected into HeLa cells, immunofluorescence staining was performed with anti-Kbz antibody. Scale bar = 10 μm.
